# Hippocampal subfield diffusion abnormalities in Parkinson’s disease with mild cognitive impairment: a volumetric and diffusion MRI study

**DOI:** 10.3389/fnagi.2026.1946629

**Published:** 2026-09-09

**Authors:** Yumei Chen, Lu Peng, Qing Hu, Ziyan Zhao, Bingchao Xu

**Affiliations:** 1 Department of Neurology, Lianyungang Clinical College of Nanjing Medical University/The First People’s Hospital of Lianyungang, Lianyungang, China; 2 Department of Neurology, The Affiliated Lianyungang Hospital of Xuzhou Medical University/The First People’s Hospital of Lianyungang, Lianyungang, China; 3 Department of Neurology, The First People’s Hospital of Lianyungang, Lianyungang, Jiangsu, China

**Keywords:** cognitive function, diffusion tensor imaging, hippocampal subfields, mild cognitive impairment, Parkinson’s disease

## Abstract

**Objective:**

To investigate whether patients with Parkinson’s disease with mild cognitive impairment exhibit hippocampal subfield-specific macrostructural and microstructural alterations and to determine whether these imaging abnormalities are associated with global cognitive performance.

**Methods:**

This retrospective cross-sectional study included 34 patients with Parkinson’s disease and mild cognitive impairment (PD-MCI), 34 patients with Parkinson’s disease and preserved cognitive function (PD-NC), and 18 healthy controls (HCs). Bilateral hippocampal subfield volumes were quantified from T1-weighted magnetic resonance images, while fractional anisotropy (FA), axial diffusivity (AD), mean diffusivity (MD), and radial diffusivity (RD) were derived from diffusion tensor imaging. Between-group differences were assessed using analysis of covariance. Diffusion models were adjusted for age, sex, and years of education, whereas volumetric models were additionally adjusted for estimated total intracranial volume. The Benjamini–Hochberg false discovery rate (FDR) procedure was applied across 182 imaging outcomes, and outcomes surviving global FDR correction were followed by Tukey-adjusted pairwise comparisons. Exploratory partial correlations assessed associations between FDR-positive diffusion measures and MoCA scores in the combined PD cohort after adjustment for age, sex, and years of education. BH-FDR correction was applied across the 42 correlation tests, and an additional sensitivity analysis further adjusted for PD-MCI/PD-NC group membership.

**Results:**

None of the 38 volumetric measures survived global FDR correction. In contrast, significant group effects were identified in 42 diffusion measures, comprising 14 AD, 2 FA, 12 MD, and 14 RD outcomes. The strongest effects for the diffusivity measures were observed in the left GC-ML-DG-head (AD: F (2,80) = 11.87, q = 0.002, partial η^2^ = 0.229; MD: F (2,80) = 11.89, q = 0.002, partial η^2^ = 0.229; RD: F (2,80) = 12.34, q = 0.002, partial η^2^ = 0.236). Tukey-adjusted pairwise comparisons identified 38 significant PD-NC versus PD-MCI contrasts and 34 HC versus PD-MCI contrasts, whereas only one HC versus PD-NC contrast was significant. This involved FA in the left GC-ML-DG-head, which was lower in both PD groups than in HC. In exploratory pooled-PD analyses, 39 of the 42 diffusion measures were associated with MoCA scores after FDR correction; however, none remained significant after additional adjustment for PD-MCI/PD-NC group membership.

**Conclusion:**

PD-MCI was characterized by distributed hippocampal diffusivity alterations despite the absence of detectable volumetric differences. Subfield-specific diffusion measures may provide complementary information on cognition-related hippocampal involvement beyond conventional volumetry, but their diagnostic and prognostic value requires longitudinal and external validation.

## 1 Introduction

Parkinson’s disease (PD) represents a progressive neurodegenerative disorder characterized not only by cardinal motor manifestations but also by a wide range of non-motor disturbances, among which cognitive impairment has emerged as a major contributor to functional decline and reduced quality of life (Aarsland et al., 2017). Cognitive deterioration in PD occurs along a clinical continuum, ranging from Parkinson’s disease with mild cognitive impairment (PD-MCI) to Parkinson’s disease dementia (PDD), with profound consequences for patients’ independence and daily functioning (Aarsland et al., 2021; Rodríguez-Antigüedad et al., 2025). Notably, approximately 30–40% of individuals with PD-MCI eventually develop dementia, emphasizing the clinical importance of identifying reliable biomarkers capable of detecting early cognitive vulnerability and predicting disease progression (Kehagia et al., 2010; Baiano et al., 2020).

The pathological basis of PD is primarily associated with the abnormal accumulation of α-synuclein and the subsequent cascade of neurodegenerative processes (Goedert et al., 2013). Importantly, α-synuclein pathology is not restricted to the nigrostriatal dopaminergic system but also extends to limbic structures, including the hippocampus, where post-mortem studies have demonstrated prominent α-synuclein deposition, particularly within the CA2 subfield (Villar-Conde et al., 2021). Experimental studies and electrophysiological investigations have demonstrated that hippocampal α-synuclein deposition may interfere with neuronal excitability, synaptic communication, and local network stability, thereby affecting hippocampus-dependent memory functions (Diógenes et al., 2012; Hall et al., 2014). Consistent with these mechanistic observations, neuropathological and neuroimaging studies have reported progressive hippocampal abnormalities accompanying cognitive worsening in PD, with more evident structural and functional alterations observed in patients with PD-MCI and PDD (Jellinger, 2023). These observations support the notion that hippocampal disruption may constitute an important neural substrate involved in the transition from PD with normal cognition (PD-NC) toward PD-MCI, and further indicate that the hippocampus represents a potential imaging target for investigating early cognitive changes in PD (Foo et al., 2017; Pan et al., 2025).

The hippocampus is a key neural substrate supporting learning and memory and is considered highly susceptible to neurodegenerative changes (Johnson, 2023). Rather than representing a uniform anatomical entity, the hippocampus consists of multiple subfields with distinct cytoarchitectural properties, connectivity patterns, and functional roles, including the CA regions, dentate gyrus, subiculum, and hippocampus–amygdala transition area (HATA). These subregions contribute differentially to cognitive processing. The CA1 region is critically involved in episodic memory retrieval (Dimsdale-Zucker et al., 2018), the dentate gyrus supports pattern separation (Chang and Hen, 2024), and the subiculum serves as the principal output region of the hippocampal formation by relaying processed information to widespread cortical and subcortical targets (Rahimi et al., 2024; Kinman et al., 2026). In contrast, HATA is a transitional subfield located at the hippocampal–amygdala junction and is anatomically positioned to participate in hippocampal–amygdala interactions (Juan et al., 2025). Accumulating evidence suggests that these subregions are differentially affected in neurodegenerative disease and may better capture disease-specific cognitive trajectories than global hippocampal measures (Foo et al., 2017; Xu et al., 2023). In PD, hippocampal atrophy is consistently associated with cognitive impairment, although it lacks disease specificity as similar patterns are observed in Alzheimer’s disease (Heim et al., 2017; Meijer et al., 2017; Cabinio et al., 2018). Recent high-resolution neuroimaging studies suggest region-specific vulnerability, with alterations in CA1, CA2–3, CA4/dentate gyrus, and the left parasubiculum, presubiculum, and HATA linked to poorer cognition and conversion from normal cognition to PD-MCI (Cabinio et al., 2018; Hrybouski et al., 2019; Xu et al., 2023).

However, most previous studies have focused primarily on volumetric alterations of hippocampal subfields. Compared with conventional structural MRI, diffusion tensor imaging (DTI) provides quantitative information regarding tissue integrity and is capable of detecting subtle microstructural abnormalities that may not be evident on structural imaging alone (Li et al., 2025). DTI enables *in vivo* assessment of tissue microstructure by quantifying water diffusion properties through metrics such as fractional anisotropy (FA), mean diffusivity (MD), axial diffusivity (AD), and radial diffusivity (RD) (Moody et al., 2022; Chen et al., 2023b). Previous studies have demonstrated that DTI-derived measures can predict cognitive decline and disease progression in PD (Chen et al., 2023a; Huang et al., 2024). DTI studies have demonstrated microstructural abnormalities in hippocampal subfields across neurodegenerative and epileptic disorders, including Alzheimer’s disease and refractory epilepsy (Chau Loo Kung et al., 2022; Shahid et al., 2022). Nevertheless, investigations of hippocampal subfield microstructure in PD-related cognitive impairment remain scarce, and whether microstructural abnormalities occur within the same subfields that exhibit volumetric loss remains unclear.

Although hippocampal atrophy and diffusion abnormalities have both been reported in PD-related cognitive impairment, most previous studies have investigated these alterations independently and primarily at the whole-hippocampus level. Consequently, it remains unclear whether macrostructural and microstructural abnormalities co-localize within specific hippocampal subfields and how these imaging alterations relate to cognitive impairment in PD-MCI. Clarifying the spatial correspondence between macrostructural and microstructural abnormalities within hippocampal subfields may provide important insights into the neurobiological mechanisms underlying cognitive decline in PD.

Therefore, the present study aimed to characterize macrostructural and microstructural alterations in hippocampal subfields in patients with PD-MCI using high-resolution structural MRI and diffusion tensor imaging. We further examined the associations between subfield-specific imaging measures and global cognitive performance. By integrating volumetric, diffusion, and cognition-related analyses at the hippocampal subfield level, this study sought to clarify hippocampal involvement in PD-MCI and identify candidate diffusion measures for further investigation of cognitive vulnerability in PD. We hypothesized that patients with PD-MCI would exhibit selective hippocampal subfield abnormalities, particularly in diffusion-derived measures, and that greater microstructural alterations would be associated with poorer cognitive performance.

## 2 Materials and methods

### 2.1 Participants

This retrospective cross-sectional study included 82 patients with Parkinson’s disease who were evaluated at the First People’s Hospital of Lianyungang between October 2023 and December 2025. Healthy controls (HC; *n* = 18) matched for age and sex were recruited from the hospital’s medical examination center during the same period. This study was conducted in accordance with the Declaration of Helsinki and approved by the Ethics Committee of the First People’s Hospital of Lianyungang (Approval No. KY-20240924001). Written informed consent was obtained from all participants prior to participation.

PD was diagnosed by two neurologists specializing in movement disorders according to the 2015 Movement Disorder Society clinical diagnostic criteria for Parkinson’s disease (Postuma et al., 2015). Participants were eligible for inclusion if they had a disease duration of ≥1 year following the initial diagnosis of PD and were able to complete neuropsychological testing and multimodal imaging assessments. Exclusion criteria were as follows: (1) missing essential clinical or imaging data; (2) secondary Parkinsonism or atypical Parkinsonian syndromes; (3) intracranial space-occupying lesions; (4) severe cerebrovascular disease, traumatic brain injury, or other neurological disorders that could affect cognitive function; (5) Hoehn–Yahr (H–Y) stage > 3 or the presence of severe motor or consciousness deficits; and (6) diagnosis of PDD.

Cognitive status was classified according to the Level I criteria proposed by the Movement Disorder Society Task Force for mild cognitive impairment in Parkinson’s disease (Litvan et al., 2012). PD-MCI was defined by evidence of cognitive decline reported by the patient, an informant, or the treating clinician; objective cognitive impairment identified using an abbreviated cognitive assessment; preserved functional independence; and absence of dementia or other conditions that could substantially interfere with cognitive performance. A Montreal Cognitive Assessment (MoCA) score <26 was used as a supportive screening threshold rather than as the sole diagnostic criterion (Dalrymple-Alford et al., 2010). The Mini-Mental State Examination (MMSE) was used to characterize global cognitive status and was not independently used to determine cognitive-group assignment. Patients who did not meet the criteria for either PD-MCI or Parkinson’s disease dementia and who showed no clinically meaningful cognitive decline were classified as PD with normal cognition (PD-NC).

Healthy controls had no history of parkinsonism, dementia, major neurological or psychiatric disorders, or other conditions likely to affect cognitive performance. They underwent the same cognitive screening and MRI quality-control procedures as the PD groups.

After screening, six patients were excluded because of incomplete baseline information and eight because of excessive head motion or inadequate MRI quality. The final sample comprised 34 patients with PD-MCI, 34 patients with PD-NC, and 18 HCs.

### 2.2 Clinical information and neuropsychological assessment

Baseline demographic and clinical characteristics, including age, sex, years of education, disease duration, and antiparkinsonian medication use, were recorded for all participants. Antiparkinsonian medication dosages were standardized as levodopa equivalent daily doses (LEDDs) according to the conversion formula recommended by the International Parkinson and Movement Disorder Society (Jost et al., 2023). Motor impairment was assessed using the Unified Parkinson’s Disease Rating Scale (UPDRS; Parts I–III), and disease severity was determined using the modified Hoehn and Yahr (H&Y) staging scale. Non-motor symptoms were evaluated using validated clinical instruments. Anxiety and depressive symptoms were assessed with the Hamilton Anxiety Rating Scale (HAMA) and the Hamilton Depression Rating Scale (HAMD), respectively, whereas sleep quality was assessed using the Pittsburgh Sleep Quality Index (PSQI). Global cognitive function was evaluated using the Mini-Mental State Examination (MMSE) and the Montreal Cognitive Assessment (MoCA).

### 2.3 MRI image acquisition

MRI data were acquired using a 3.0-T Siemens MAGNETOM Prisma scanner (Siemens Healthineers, Erlangen, Germany) equipped with a 64-channel head-and-neck coil. Patients with Parkinson’s disease were instructed to withhold their antiparkinsonian medications for at least 24 h before MRI acquisition and were scanned in a practically defined OFF-medication state. This interval was used to minimize the acute effects of dopaminergic medication during imaging; however, it was not intended to represent complete pharmacological washout of all antiparkinsonian agents. High-resolution three-dimensional T1-weighted images were acquired in the sagittal plane using a gradient-echo inversion-recovery sequence with the following parameters: repetition time/echo time/inversion time = 2300/3/900 ms, flip angle = 9°, field of view = 256 × 256 mm^2^, acquisition matrix = 256 × 256, number of slices = 192, slice thickness = 1.0 mm, and voxel size = 1.0 × 1.0 × 1.0 mm^3^. Diffusion-weighted images were acquired using an axial single-shot spin-echo echo-planar imaging sequence with the following parameters: repetition time/echo time = 9300/69 ms, flip angle = 90°, field of view = 256 × 256mm^2^, acquisition matrix = 104 × 104, number of slices = 66, slice thickness = 2.0 mm, and voxel size = 2.0 × 2.0 × 2.0 mm^3^. Diffusion sensitization was applied along 64 non-collinear directions at a b value of 1000 s/mm^2^, together with one non-diffusion-weighted image acquired at b = 0 s/mm^2^. No additional b = 0 image with reversed phase-encoding polarity was acquired; therefore, susceptibility-induced EPI distortion correction using FSL TOPUP could not be performed. Two signal averages were obtained. The diffusion acquisition time was approximately 6 min 32 s, and whole-brain coverage was achieved. Before image processing, all structural and diffusion-weighted images were independently reviewed by two neuroimaging specialists with more than 5 years of experience who were blinded to the participants’ cognitive-group assignments. Images were evaluated for completeness, head motion, signal dropout, ghosting, susceptibility-related distortion, and other major artifacts. Datasets showing severe motion artifacts, missing image volumes, inadequate whole-brain coverage, or insufficient signal-to-noise ratio were excluded from subsequent analyses.

### 2.4 Image processing

Image processing was performed using MRIcroGL for DICOM-to-NIfTI conversion, FreeSurfer 7.4.1^1^ for structural processing and hippocampal subfield segmentation, MRtrix3 3.0.4^2^ and FSL 6.0.7.17^3^ for diffusion preprocessing and tensor estimation, and ANTs^4^ for N4 bias-field correction.

Structural and diffusion data were converted from DICOM to NIfTI format using MRIcroGL. The 3D T1-weighted images were processed using the FreeSurfer automated pipeline, including skull stripping, intensity normalization, spatial normalization, and anatomical segmentation. Hippocampal subfield segmentation was subsequently performed using the FreeSurfer probabilistic atlas based on the T1-weighted images alone; no dedicated high-resolution T2-weighted sequence was available for subfield segmentation. The bilateral segmentation included 19 hippocampal regions: presubiculum-head, subiculum-head, CA1-head, CA3-head, CA4-head, granule cell and molecular layer of the dentate gyrus (GC-ML-DG)-head, molecular_layer_HP-head, presubiculum-body, subiculum-body, CA1-body, CA3-body, CA4-body, GC-ML-DG-body, molecular_layer_HP-body, parasubiculum, hippocampus–amygdala transition area (HATA), fimbria, hippocampal-fissure, and hippocampal-tail. Volumetric measurements were extracted for all segmented hippocampal regions. For diffusion analyses, the hippocampal-fissure was excluded because it primarily represents a cerebrospinal fluid (CSF)-containing anatomical space rather than neural tissue and is particularly susceptible to partial-volume effects. Diffusion metrics were therefore calculated for the remaining 18 hippocampal regions in each hemisphere. In addition, volumes of the whole hippocampal head and body were extracted, together with the estimated total intracranial volume (eTIV). For additional global structural characterization, total bilateral hippocampal volume was calculated as the sum of the left and right whole-hippocampus volumes derived from the FreeSurfer segmentation outputs. Estimated total intracranial volume (eTIV) was retained as the measure of intracranial size used for volumetric adjustment.

**FIGURE 1:**
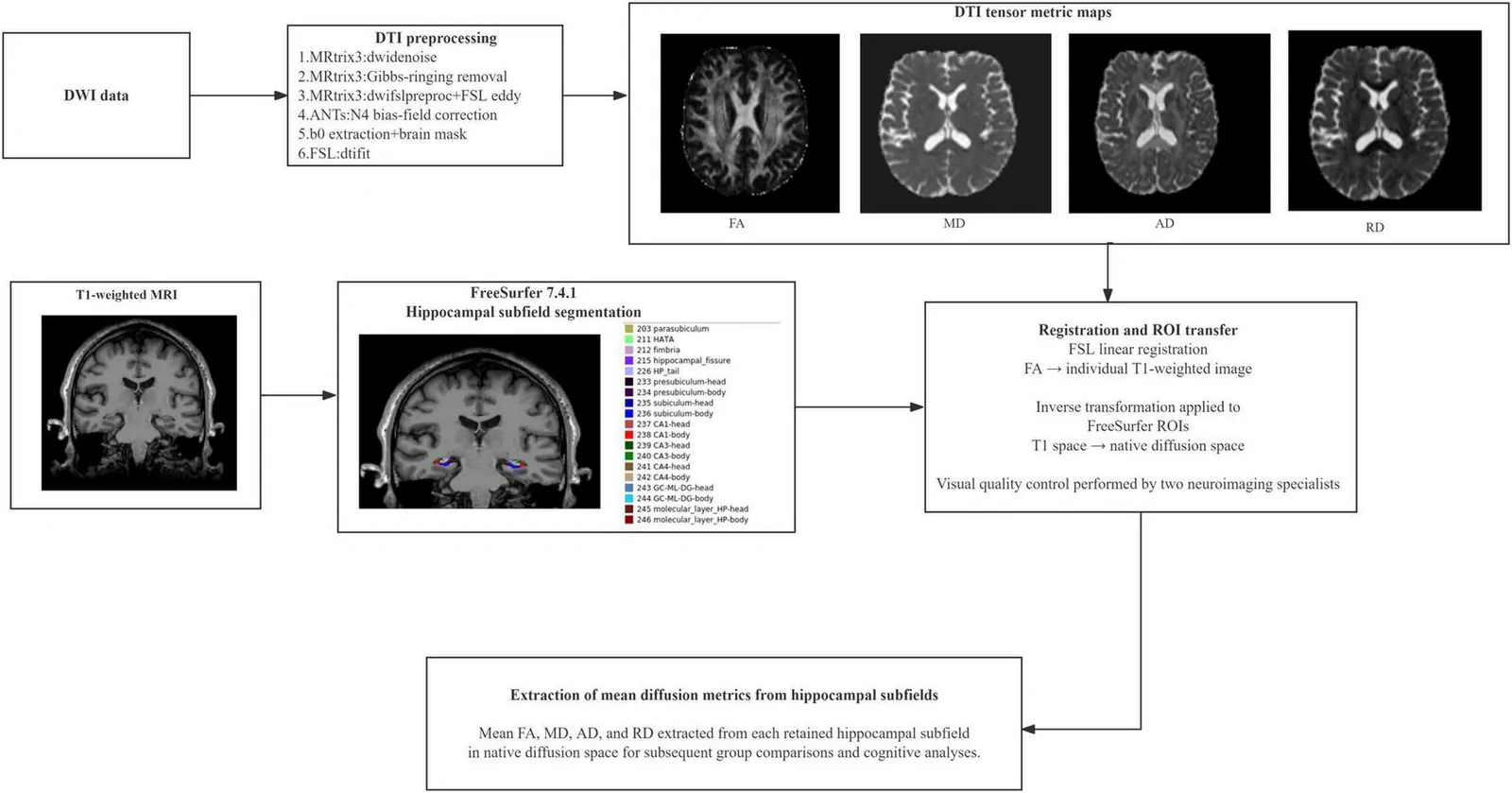
Workflow of hippocampal subfield segmentation, diffusion preprocessing, registration, and ROI-based diffusion metric extraction.

Diffusion-weighted images were first denoised using the MRtrix3 dwidenoise function, followed by Gibbs-ringing removal using the MRtrix3 mrdegibbs function. Subject motion and eddy-current-induced distortions were then corrected using FSL EDDY through the MRtrix3 dwifslpreproc workflow, with the diffusion gradient directions (b-vectors) rotated accordingly based on the estimated motion parameters. Because no reversed phase-encoding b = 0 pair was available, susceptibility-induced EPI distortions were not explicitly corrected using FSL TOPUP. Consequently, residual geometric distortion may have remained in the diffusion images and could have reduced the local accuracy of diffusion-to-T1 registration, particularly in small hippocampal regions. N4 bias-field correction was subsequently performed using ANTs. A b = 0 image was extracted and used to generate the brain mask. Diffusion tensors were fitted using FSL dtifit to derive fractional anisotropy (FA), mean diffusivity (MD), axial diffusivity (AD), and radial diffusivity (RD) maps. The FA image was linearly registered to each participant’s T1-weighted anatomical image using FSL. The resulting transformation was inverted and applied to the FreeSurfer-derived hippocampal masks to map the ROIs into native diffusion space. Because residual susceptibility-related distortion could affect local registration accuracy, all transformed hippocampal ROIs were visually reviewed in native diffusion space by two neuroimaging specialists for anatomical plausibility, registration accuracy, and obvious overlap with CSF-containing regions. Mean FA, MD, AD, and RD values were then extracted from each retained hippocampal subfield in native diffusion space and exported for subsequent statistical analysis. The overall image-processing, hippocampal subfield segmentation, registration, ROI-transfer, and diffusion-metric extraction workflow is summarized in Figure 1.

### 2.5 Statistical analysis

Analyses were performed in R 4.5.1 and GraphPad Prism 10.4.1. Continuous variables were assessed using the Shapiro–Wilk test and Levene’s test. Data are summarized as mean ± SD when all three groups were approximately normally distributed and as median (Q1, Q3) when at least one group was non-normal. Categorical variables are reported as n (%). Baseline comparisons used one-way ANOVA, Kruskal–Wallis tests, Chi-square tests, or Fisher’s exact tests, as appropriate. Significant omnibus tests were followed by Bonferroni-adjusted parametric contrasts or Dunn–Bonferroni tests. PD-specific variables were compared between PD-NC and PD-MCI using independent-samples *t*-tests, Mann–Whitney *U* tests, or exact tests, as appropriate.

Separate ANCOVA models were fitted for 38 volumetric outcomes and 36 outcomes within each diffusion metric (FA, AD, MD, and RD), yielding 182 imaging outcomes in total. Group was entered as the fixed factor. Diffusion models were adjusted for age, sex, and years of education, whereas volumetric models were additionally adjusted for estimated total intracranial volume (eTIV). For the primary analysis, Benjamini–Hochberg false discovery rate (BH-FDR) correction was applied across all 182 omnibus group tests, with q < 0.05 considered statistically significant. To avoid excessive multiplicity correction, only outcomes surviving the global FDR procedure were subsequently examined using Tukey-adjusted pairwise comparisons of estimated marginal means among the PD-MCI, PD-NC, and HC groups, with 95% confidence intervals reported. Given the nonsignificant volumetric findings, a sensitivity analysis was additionally performed to evaluate the ability of the present sample to detect a small-to-moderate between-group volumetric effect. At a two-sided α of 0.05, statistical power was estimated for a standardized effect size of Cohen’s d = 0.50, and the minimum detectable effect size corresponding to 80% power was calculated for each pairwise group comparison. All tests were two-sided, with *P* < 0.05 considered statistically significant unless otherwise specified.

As a secondary exploratory analysis, the 42 diffusion outcomes that survived global FDR correction in the primary group comparisons were examined for associations with MoCA scores in the combined PD cohort (*n* = 68). Partial correlations were adjusted for age, sex, and years of education. The resulting 42 *P* values were jointly corrected using the Benjamini–Hochberg FDR procedure, with q < 0.05 considered statistically significant. Because MoCA contributed to the clinical characterization of cognitive status, an additional sensitivity analysis was performed with PD-MCI/PD-NC group membership included as an additional covariate to assess whether the observed associations persisted beyond categorical cognitive-group differences. The 42 *P* values from this sensitivity analysis were separately subjected to BH-FDR correction. These analyses were considered exploratory and were not interpreted as independent validation of the categorical group comparisons.

## 3 Results

### 3.1 Demographics and neuropsychological tests of the participants

**FIGURE 2:**
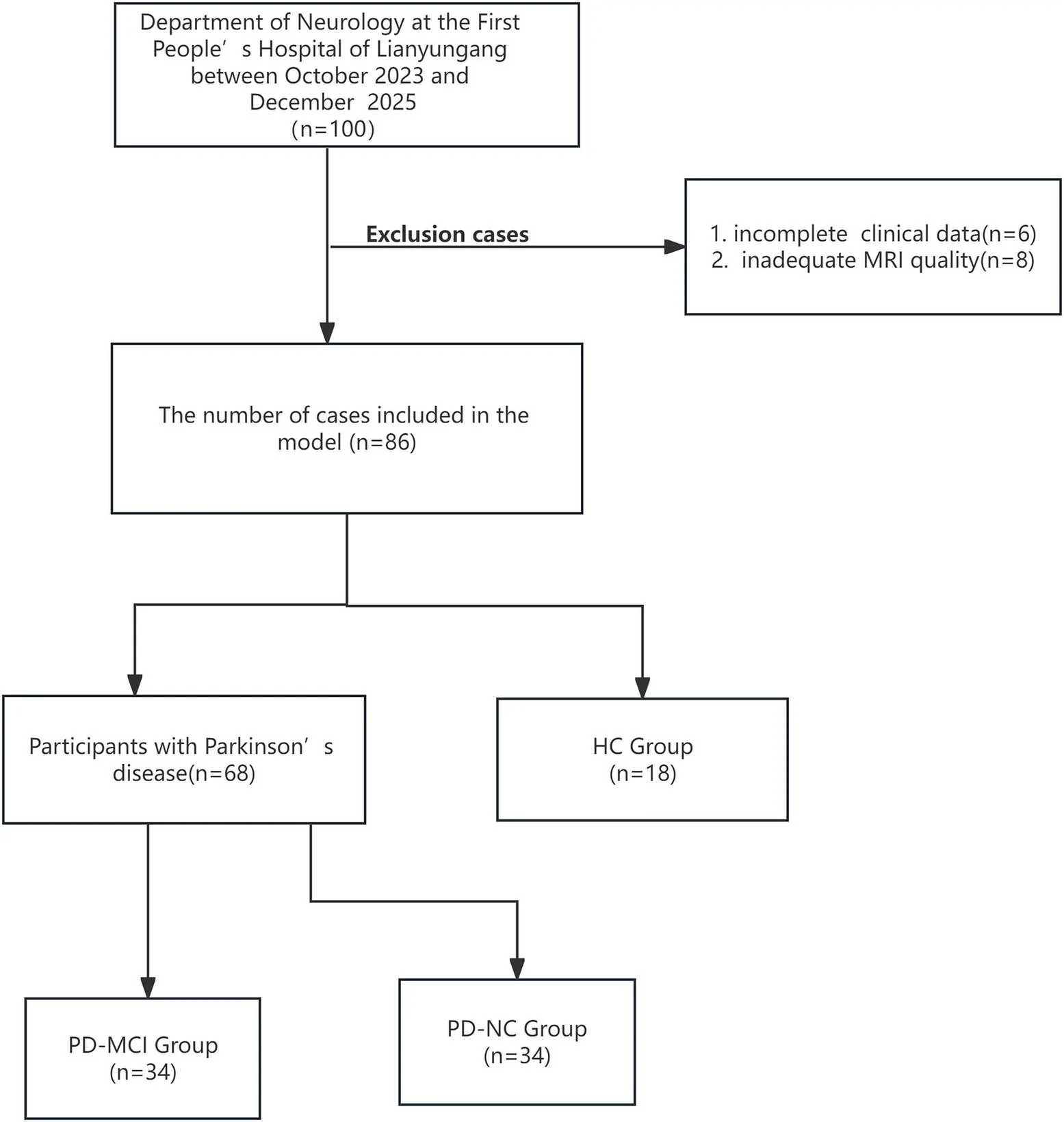
Participant selection and group allocation.

The final analysis included 34 patients with PD-MCI, 34 patients with PD-NC, and 18 HC (Figure 2).

Demographic and clinical characteristics are presented in Table 1. No significant differences were found among the three groups in age, sex, PSQI scores, HAMA scores, HAMD scores, or eTIV (all *P* > 0.05). Educational duration differed significantly among groups (*P* = 0.044). Compared with PD-NC and HC, patients with PD-MCI exhibited significantly lower MoCA and MMSE scores (both *P* < 0.001). Within the PD cohort, no significant differences were observed in disease duration, Hoehn–Yahr stage, UPDRS scores, or LEDD between PD-MCI and PD-NC groups (all *P* > 0.05). As an additional global structural measure, total bilateral hippocampal volume was compared across groups after adjustment for age and eTIV. No significant group effect was detected (F (2,81) = 0.225, *P* = 0.799, partial η^2^ = 0.006).

**TABLE 1:** Comparison of demographic and clinical characteristics among patients with Parkinson’s disease with mild cognitive impairment, Parkinson’s disease with normal cognition, and healthy controls.

Variable	PD-MCI (*n* = 34)	PD-NC (*n* = 34)	HC (*n* = 18)	*P*
Age, years	68.97 ± 5.88	65.97 ± 6.30	67.78 ± 6.58	0.140
Sex, male (n %)	17 (50.0)	23 (67.6)	8 (44.4)	0.188
Education, years	10.44 ± 2.51	11.82 ± 3.12	9.78 ± 3.59	0.044
MoCA	20 (19,22)	28 (27,29)^a^	29 (28,30)^a^	<0.001
MMSE	23 (20,25)	29 (28,30)^a^	30 (29,30)^a^	<0.001
HAMA	6 (4,11)	8 (7,10)	8 (7,9)	0.063
HAMD	7 (4,10)	9 (7,10)	8 (8,9)	0.079
PSQI	8.18 ± 4.11	6.97 ± 2.93	6.67 ± 1.46	0.104
eTIV ( × 10^3^ mm^3^)	1461.30 ± 193.05	1564.11 ± 202.81	1491.21 ± 137.44	0.076
Total bilateral hippocampal volume (mm^3^)	6796.28 ± 960.45	7244.50 ± 801.45	7041.16 ± 852.38	0.799*
Disease duration, years	2 (1.38,3.25)	2 (1.38,3.00)	–	0.398
H&Y Stage *n* (%)				0.445
Stage 1	12 (35.3)	15 (44.1)	–	
Stage 2	17 (50.0)	17 (50.0)	–	
Stage 3	5 (14.7)	2 (5.9)	–	
LEDD (mg/day)	533.09 ± 99.19	550.74 ± 68.94	–	0.397
UPDRS-I	8.06 ± 3.69	8.38 ± 3.35	–	0.706
UPDRS-II	13.53 ± 4.32	14.26 ± 4.81	–	0.509
UPDRS-III	25.68 ± 7.62	22.35 ± 7.91	–	0.082

Data are expressed as mean ± SD, median (IQR), or count (%). Group comparisons were performed using one-way analysis of variance (ANOVA), Kruskal–Wallis test, or Chi-square test, as appropriate. *Post hoc* comparisons were performed using Bonferroni correction when applicable. Superscripts indicate significant pairwise differences:

*^a^p* < 0.05 vs. PD-MCI group. PD, Parkinson’s disease; PD-MCI, Parkinson’s disease with mild cognitive impairment; PD-NC, Parkinson’s disease with normal cognition; HC, healthy control; MoCA, Montreal Cognitive Assessment; MMSE, Mini-Mental State Examination; HAMA, Hamilton Anxiety Rating Scale; HAMD, Hamilton Depression Rating Scale; PSQI, Pittsburgh Sleep Quality Index; eTIV, estimated total intracranial volume; H&Y, Hoehn–Yahr stage; LEDD, levodopa equivalent daily dose; UPDRS, Unified Parkinson’s Disease Rating Scale.

*Group differences in total bilateral hippocampal volume were assessed using ANCOVA adjusted for age and eTIV.

### 3.2 Volumetric findings and sensitivity analysis

After adjustment for age, sex, years of education, and eTIV, none of the 38 hippocampal volumetric measures showed a significant group effect after global FDR correction. The smallest unadjusted *P* value among the volumetric outcomes was 0.139 for the left fimbria (q = 0.324).

Because the absence of significant volumetric differences could reflect limited statistical sensitivity, an additional power/sensitivity analysis was performed. With 34 participants in each PD group, the estimated power to detect a standardized between-group difference of Cohen’s d = 0.50 at a two-sided α = 0.05 was 52.9%, and the effect size required to achieve 80% power was approximately d = 0.690. For comparisons between either PD group (n = 34) and HCs (n = 18), the corresponding power for d = 0.50 was 39.1%, with a minimum detectable effect of approximately d = 0.833 for 80% power. These findings indicate that the present sample had limited sensitivity to small-to-moderate volumetric differences.

### 3.3 Microstructural alterations of hippocampal subfields

Diffusion measures showed substantially more group-related differences than volumetric measures. A summary of the globally FDR-positive hippocampal imaging outcomes and Tukey-adjusted pairwise comparisons is presented in Table 2. After global BH-FDR correction across all 182 imaging outcomes, 42 diffusion measures remained significant, including 14 AD, 2 FA, 12 MD, and 14 RD measures. The strongest effects for the diffusivity metrics were consistently observed in the left GC-ML-DG-head (AD: F = 11.87, q = 0.002, partial η^2^ = 0.229; MD: F = 11.89, q = 0.002, partial η^2;^ = 0.229; RD: F = 12.34, q = 0.002, partial η^2^ = 0.236). Other significant abnormalities involved the bilateral subiculum, GC-ML-DG, fimbria, and CA1-related regions, as well as the left CA3, HATA, molecular layer, and CA4.

**TABLE 2:** Summary of globally FDR-positive hippocampal imaging outcomes and Tukey-adjusted pairwise comparisons.

Metric	Global FDR-positive outcomes	PD-NC vs. PD-MCI	HC vs. PD-MCI	HC vs. PD-NC
Volume	0/38	–	–	–
FA	2/36	1	2	1
AD	14/36	13	11	0
MD	12/36	12	9	0
RD	14/36	12	12	0

q values were obtained using the Benjamini–Hochberg FDR procedure across all 182 imaging outcomes. Pairwise counts represent Tukey-adjusted comparisons with *P* < 0.05 among outcomes surviving global FDR correction. Diffusion models were adjusted for age, sex, and years of education; volumetric models were additionally adjusted for eTIV.

**FIGURE 3:**
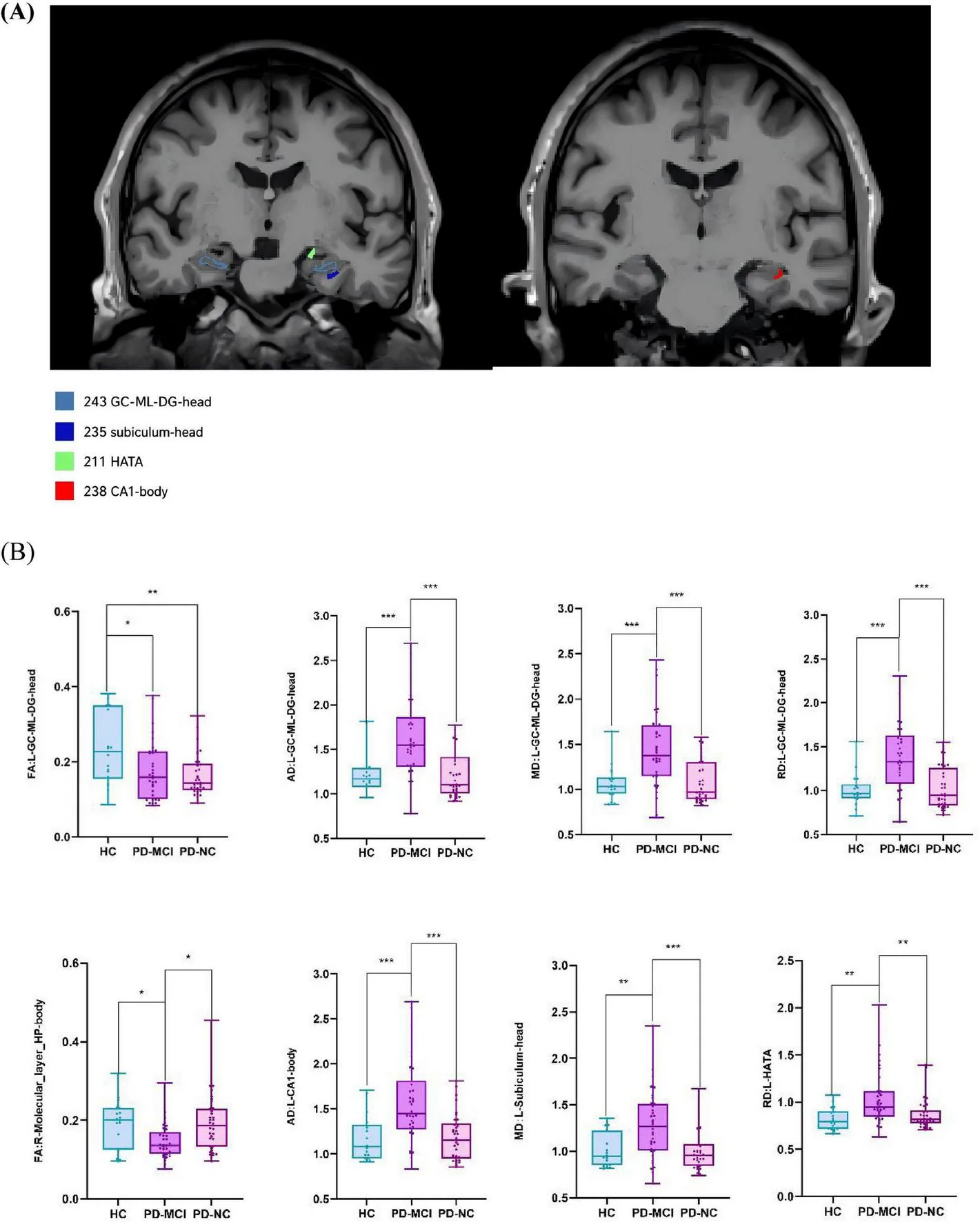
Anatomical localization and representative diffusion abnormalities of hippocampal subfields across groups. **(A)** Representative coronal T1-weighted images showing the anatomical localization of selected hippocampal subfields that exhibited significant group effects in diffusion measures after global Benjamini–Hochberg false discovery rate (FDR) correction. The highlighted regions include the GC-ML-DG-head, subiculum-head, HATA, and CA1-body. These regions are shown as representative FDR-positive ROIs rather than an exhaustive display of all significant subfields. **(B)** Boxplots show unadjusted individual observations, with boxes representing the median and interquartile range. Statistical brackets indicate significant Tukey-adjusted pairwise comparisons of estimated marginal means for outcomes surviving global Benjamini–Hochberg FDR correction. Diffusion models were adjusted for age, sex, and years of education. *P < 0.05, **P < 0.01, and ***P ≤ 0.001. FA, fractional anisotropy; AD, axial diffusivity; MD, mean diffusivity; RD, radial diffusivity; GC-ML-DG, granule cell and molecular layer of the dentate gyrus; HATA, hippocampus–amygdala transition area; HC, healthy controls; PD-NC, Parkinson’s disease with normal cognition; PD-MCI, Parkinson’s disease with mild cognitive impairment. AD, MD, and RD are expressed in × 10^−3^ mm^2^/s; FA is dimensionless.

Among the 42 FDR-positive diffusion outcomes, Tukey-adjusted pairwise comparisons identified 73 significant contrasts. Of these, 38 involved PD-NC versus PD-MCI, 34 involved HC versus PD-MCI, and only one involved HC versus PD-NC. No AD, MD, or RD measure showed a significant HC versus PD-NC difference after Tukey adjustment. The only significant HC versus PD-NC contrast was observed for FA in the left GC-ML-DG-head, for which adjusted FA was higher in HC than in both PD-NC and PD-MCI, whereas PD-NC and PD-MCI did not differ significantly. Thus, the pairwise pattern did not support a generalized intermediate diffusion phenotype in PD-NC. The anatomical localization of selected FDR-positive hippocampal subfields, together with representative diffusion measures showing significant between-group differences, is presented in Figure 3.

### 3.4 Exploratory associations between hippocampal diffusion measures and cognitive performance

As an exploratory analysis complementary to the categorical group comparisons, the 42 diffusion outcomes that survived the primary global FDR screen were examined for associations with MoCA scores in the combined PD cohort. After adjustment for age, sex, and years of education, 39 of the 42 diffusion measures remained significantly associated with MoCA after BH-FDR correction. Most AD, MD, and RD measures showed negative associations with MoCA, with the strongest association observed for AD in the left CA1-body (partial r = -0.496, q < 0.001). Because MoCA contributed to the clinical characterization of cognitive status, an additional sensitivity analysis was performed with PD-MCI/PD-NC group membership included as an additional covariate. After adjustment for group membership and BH-FDR correction across the 42 tests, none of the imaging–MoCA associations remained statistically significant (all q ≥ 0.264). These findings indicate that the pooled continuous associations were substantially influenced by between-group differences in cognitive status and should therefore be interpreted as exploratory rather than as independent confirmation of the categorical ANCOVA findings. Additional supporting results are provided in Supplementary material 1.

## 4 Discussion

This study investigated hippocampal subfield volumes and diffusion characteristics in patients with PD-MCI, patients with PD-NC, and healthy controls. Several main findings emerged. First, no hippocampal subfield volume showed a significant group difference after global FDR correction, whereas multiple AD, MD, and RD measures showed significant group effects, predominantly reflecting higher diffusivity in PD-MCI relative to the other groups. Second, HC and PD-NC were largely similar for diffusivity measures; however, FA in the left GC-ML-DG-head was significantly lower in both PD groups than in HC. Thus, the pairwise findings do not support a generalized intermediate diffusion phenotype in PD-NC. Third, although widespread associations between higher diffusivity and lower MoCA scores were observed in the pooled PD cohort, none remained significant after additional adjustment for PD-MCI/PD-NC group membership and FDR correction, indicating that these exploratory associations were substantially influenced by between-group cognitive-status differences.

The dissociation between volumetric and diffusion findings should be interpreted with caution. Diffusion measures characterize the mobility and directional dependence of water within tissue but do not provide pathologically specific markers of cellular injury (Alexander et al., 2007; Jones et al., 2013). This distinction is particularly important in hippocampal gray matter, where conventional white-matter interpretations of RD as a marker of demyelination and AD as a marker of axonal injury cannot be directly extrapolated. Diffusion MRI is sensitive to variations in neurite and dendritic architecture within the hippocampus, as demonstrated by diffusion–histology studies of hippocampal neurite loss (Vestergaard-Poulsen et al., 2011), and gray-matter diffusion measures are also influenced by underlying cellular density and cytoarchitecture (Baxi et al., 2022). Furthermore, experimental studies have demonstrated sensitivity of diffusion MRI to microglial and astrocytic activation and neuroinflammatory processes (Garcia-Hernandez et al., 2022). Consistent with this nonspecific interpretation, increased AD, MD, and RD in the hippocampus have also been reported in association with inflammation and reactive astrogliosis in experimental brain injury (Constanzo et al., 2018). Accordingly, the higher diffusivity observed in the present study is more appropriately interpreted as altered tissue organization or reduced structural barriers to water diffusion rather than direct evidence of a specific axonal or myelin pathology.

Previous DTI studies have reported diffusion abnormalities across cognitive stages in Parkinson’s disease and other neurodegenerative conditions (Melzer et al., 2013; Amlien and Fjell, 2014). However, these studies largely focused on white-matter alterations and do not establish that hippocampal diffusion abnormalities temporally precede volumetric atrophy. In the present study, multiple diffusion measures showed significant group effects, whereas no volumetric outcome survived global FDR correction. This pattern indicates that diffusion MRI detected group-related tissue differences that were not detected by conventional volumetry in the current sample. Nevertheless, given the limited statistical power of the volumetric analyses and the cross-sectional design, the present findings cannot establish that microstructural abnormalities precede macroscopic atrophy.

Among all hippocampal subfields examined, the GC-ML-DG showed some of the most consistent group-related diffusion abnormalities. PD-MCI patients demonstrated increased AD, MD, and RD values within the GC-ML-DG. In pooled exploratory analyses, several of these diffusion measures were associated with lower MoCA scores; however, these associations did not remain significant after additional adjustment for cognitive-group membership and FDR correction. Thus, the present data support robust group-related GC-ML-DG diffusion differences, whereas evidence for an independent continuous association with cognitive performance is less conclusive. The dentate gyrus plays a critical role in hippocampal-dependent cognition, particularly pattern separation, memory discrimination, and formation of distinct memory representations (Yassa and Stark, 2011). Dysfunction of the dentate gyrus may impair the ability to distinguish similar experiences and contribute to early cognitive vulnerability. The selective involvement of GC-ML-DG observed in our study is consistent with previous hippocampal subfield research in PD. Importantly, Foo et al. investigated hippocampal subfield alterations in PD and reported that volume reductions in GC-DG, CA4, parasubiculum, and HATA were associated with cognitive decline progression (Foo et al., 2017). However, previous investigations mainly focused on volumetric alterations. Together with previous volumetric evidence implicating the dentate gyrus in PD-related cognitive decline, the present findings suggest that the GC-ML-DG may represent a particularly vulnerable component of hippocampal circuitry in PD-MCI.

In addition to the dentate gyrus, significant microstructural abnormalities were observed in CA1 and subiculum regions, supporting the concept of selective hippocampal vulnerability in PD-MCI. CA1 represents a key computational hub within hippocampal circuitry and integrates information from upstream hippocampal regions before transmitting signals to cortical networks (Lim and Lee, 2026). Due to its high metabolic demand, extensive synaptic connectivity, and vulnerability to pathological stress, CA1 is considered one of the most susceptible hippocampal subfields in neurodegenerative disorders (Alldred et al., 2024). Previous studies have demonstrated that CA1 dysfunction is closely associated with episodic memory impairment and cognitive decline (La et al., 2019). Consistent with these findings, our study identified significant group-related diffusion abnormalities in CA1. Although CA1-body diffusion measures showed strong associations with MoCA in the pooled PD analysis, these associations did not survive FDR correction after additional adjustment for cognitive-group membership. The subiculum serves as the major output structure of the hippocampal formation and mediates communication between hippocampal circuits and widespread cortical and limbic regions (Matsumoto et al., 2019). Similarly, subiculum-head diffusion measures showed group-related abnormalities and nominal continuous associations with MoCA; however, the latter did not remain significant in the group-adjusted FDR-corrected sensitivity analysis.

The HATA was another important region affected in PD-MCI patients. Anatomically, HATA represents a transitional region between the hippocampus and amygdala and participates in memory processing, emotional regulation, and limbic network integration (Sedwick and Autry, 2022). In the present study, the left HATA demonstrated significant group-related increases in AD, MD, and RD. Exploratory pooled analyses also suggested associations with MoCA performance, although these associations did not remain significant after additional adjustment for cognitive-group membership and FDR correction. Given that cognitive impairment in PD reflects dysfunction of distributed neural networks rather than isolated regional abnormalities, disruption of HATA-related limbic circuits may contribute to broader network-level alterations associated with cognitive decline (Lenka et al., 2018).

An additional consideration concerns the imaging profile of the PD-NC group. In the present cohort, PD-NC participants had a relatively short median disease duration of 2 years, and 32 of 34 patients (94.1%) were classified as Hoehn–Yahr stage 1 or 2, indicating that the group was predominantly characterized by mild motor-stage disease. Their disease duration, Hoehn–Yahr stage, UPDRS scores, and LEDD did not differ significantly from those of the PD-MCI group. Thus, the relative similarity between PD-NC and HC for most hippocampal diffusion measures should not be interpreted as evidence that PD-NC represents a biologically “benign” subtype. Rather, it may reflect relatively limited hippocampal involvement at this disease stage, heterogeneity in the timing of cognitive-related neurodegeneration, and/or limitations of the present conventional DTI acquisition in detecting subtle early abnormalities, particularly given the 2-mm isotropic diffusion resolution and susceptibility to partial-volume and registration effects. Importantly, the revised pairwise analysis identified lower FA in the left GC-ML-DG-head in PD-NC than in HC, whereas PD-NC and PD-MCI did not differ significantly for this measure. This finding suggests that at least some hippocampal diffusion abnormalities may already be present in cognitively normal PD and may reflect PD-related tissue alterations that are not specifically linked to cognitive impairment. In contrast, the widespread increases in AD, MD, and RD were predominantly observed in PD-MCI relative to both comparison groups. Longitudinal follow-up will be required to determine whether subtle diffusion abnormalities in PD-NC are associated with subsequent cognitive decline.

The absence of significant volumetric differences also contrasts with previous studies reporting volume loss in the GC-DG, CA4, parasubiculum, HATA, and other hippocampal subfields in patients with PD-related cognitive impairment (Foo et al., 2017). This discrepancy may reflect differences in disease stage, sample composition, structural image resolution, subfield segmentation procedures, covariate selection, and multiple-comparison correction. In addition, because the spatial resolution of the diffusion images (2 × 2 × 2 mm^3^) was lower than that of the T1-weighted images (1 × 1 × 1 mm^3^), diffusion measurements in small hippocampal subfields may have been affected by registration uncertainty and partial-volume contamination. This issue is particularly relevant for subfields adjacent to CSF-containing spaces, because inclusion of CSF within a diffusion voxel would be expected to bias MD upward owing to the substantially higher diffusivity of CSF relative to hippocampal tissue. Accordingly, subtle local morphological differences and CSF partial-volume contamination may have contributed to some of the observed increases in diffusivity. Therefore, the present diffusion findings should be interpreted as reflecting altered hippocampal microstructural properties that may also be influenced by partial-volume effects, rather than as direct evidence of specific cellular injury or a temporal sequence preceding volume loss.

From a clinical perspective, the value of the present findings lies in identifying a pattern of hippocampal subfield diffusion abnormalities associated with cognitive status in PD. Diffusion measures derived from the GC-ML-DG, CA1, subiculum, and HATA may therefore represent candidate imaging measures for further investigation of cognitive vulnerability in this population. Nevertheless, the current results should not be interpreted as establishing diagnostic or prognostic biomarkers, because the study was cross-sectional, involved a relatively modest single-center sample, and lacked external validation. Larger multicenter studies with comprehensive domain-specific neuropsychological assessments and longitudinal follow-up are required to determine whether these measures can improve cognitive risk stratification or predict conversion from PD-NC to PD-MCI and Parkinson’s disease dementia.

Several limitations should be acknowledged in the present study. First, the cross-sectional design prevents determination of causal relationships between hippocampal microstructural alterations and cognitive decline. Longitudinal studies are needed to clarify whether these abnormalities represent predictive biomarkers. Second, the sample size was relatively modest, especially for the HC group. Larger multicenter studies are required to validate the reproducibility and generalizability of these findings. Third, hippocampal subfield segmentation was based on the 1-mm isotropic T1-weighted images alone, because no dedicated high-resolution T2-weighted sequence was acquired. The limited internal contrast of conventional T1-weighted imaging may reduce the precision with which boundaries between small hippocampal subfields can be delineated, increasing reliance on probabilistic atlas priors. This may have introduced segmentation uncertainty into both the subfield volumetric measurements and the diffusion analyses, because the FreeSurfer-derived subfield masks were subsequently transferred into native diffusion space for extraction of DTI metrics. Future studies incorporating dedicated high-resolution T2-weighted imaging may improve anatomical delineation of internal hippocampal subfields. Fourth, conventional DTI metrics provide indirect measures of tissue organization and lack pathological specificity, particularly in gray-matter structures such as the hippocampus. Accordingly, increased AD, MD, or RD should not be interpreted as direct evidence of axonal injury or demyelination. These changes may arise from multiple alterations in the cellular and extracellular microenvironment, including reduced neurite or dendritic complexity, altered cellular density, increased extracellular water, and glial or inflammatory responses. In addition, because no reversed phase-encoding b = 0 image was acquired, susceptibility-induced EPI distortions could not be explicitly corrected using TOPUP. Residual geometric distortion may therefore have reduced the local accuracy of diffusion-to-T1 registration and ROI transfer, particularly in small hippocampal subfields adjacent to CSF. Although all transformed ROIs were visually reviewed for anatomical plausibility and registration accuracy, this procedure cannot completely eliminate residual distortion-related measurement error. Furthermore, the 2-mm isotropic diffusion resolution increases susceptibility to partial-volume contamination in small hippocampal subfields. For regions adjacent to CSF, such contamination would be expected to bias MD upward because CSF has substantially higher diffusivity than hippocampal tissue. Although the hippocampal fissure was excluded and ROIs with obvious CSF overlap were visually checked, residual CSF partial-volume effects cannot be completely excluded. Advanced diffusion models with higher spatial resolution, together with molecular, fluid, or histopathological markers, will be required to clarify the biological substrates of these findings. Fifth, although cognitive classification was based on MDS criteria, comprehensive domain-specific neuropsychological assessments were limited. In addition, because MoCA contributed to the clinical characterization of PD-MCI versus PD-NC, pooled correlations between MoCA and diffusion measures were not fully independent of cognitive-group assignment. Although we addressed this issue using a group-adjusted sensitivity analysis, no association survived FDR correction after accounting for group membership. The cognition–imaging correlations should therefore be regarded as exploratory. Future studies should investigate whether hippocampal subfield abnormalities are associated with specific cognitive domains. Finally, although years of education were included as a covariate in the imaging analyses, education is only an indirect proxy for cognitive reserve, and residual confounding related to interindividual differences in reserve cannot be excluded. Accordingly, part of the between-group differences in cognitive performance and their associated imaging patterns may have been influenced by cognitive reserve rather than neurodegenerative processes alone. Potential effects of other disease-related factors, including antiparkinsonian medication exposure, disease duration, mood symptoms, and vascular burden, could also not be completely excluded. Although patients were instructed to withhold antiparkinsonian medications for at least 24 h before MRI acquisition, this interval does not ensure complete pharmacological washout, particularly for longer-acting agents. Therefore, residual medication-related effects cannot be entirely excluded. Future studies should incorporate these variables to further clarify the independent contribution of hippocampal microstructural abnormalities to cognitive impairment in Parkinson’s disease.

## 5 Conclusion

PD-MCI was associated with widespread increases in hippocampal subfield diffusivity, whereas no hippocampal volumetric measure survived correction for multiple comparisons. The most consistent abnormalities involved the GC-ML-DG, CA1, subiculum, and HATA. Although higher diffusivity was associated with lower MoCA scores in pooled exploratory analyses, these associations did not remain significant after additional adjustment for cognitive-group membership, indicating that they were substantially influenced by categorical cognitive-status differences. These results indicate that diffusion measures may provide information about hippocampal involvement in PD-MCI that is not captured by conventional volumetry. Longitudinal studies using larger samples, detailed neuropsychological assessment, and higher-resolution diffusion methods are required to establish the temporal and clinical significance of these findings.

## Data Availability

The original contributions presented in the study are included in the article/Supplementary material, further inquiries can be directed to the corresponding author.

## Footnotes

1
https://surfer.nmr.mgh.harvard.edu

2
https://www.mrtrix.org

3
https://fsl.fmrib.ox.ac.uk

4
https://antsx.github.io/ANTs
