# Supplementary material for: Hippocampal subfield diffusion abnormalities in Parkinson’s disease with mild cognitive impairment: a volumetric and diffusion MRI study
Source: Front Aging Neurosci. 2026 Sep 9;18:1946629. doi: 10.3389/fnagi.2026.1946629 (PMC13598964; doi:10.3389/fnagi.2026.1946629)
Supplement: Supplementary file 1 [file Data_Sheet_1.pdf]

Table 1. Complete omnibus group-effect tests for all 182 imaging outcomes.

Type	Outcome	F	P	Global FDR q	Partial η²
RD	L-GC-ML-DG-head	12.34	<0.001	0.002	0.236
MD	L-GC-ML-DG-head	11.89	<0.001	0.002	0.229
AD	L-GC-ML-DG-head	11.87	<0.001	0.002	0.229
RD	R-GC-ML-DG-head	11.16	<0.001	0.002	0.218
AD	L-CA3-body	10.68	<0.001	0.002	0.211
MD	R-GC-ML-DG-head	10.66	<0.001	0.002	0.210
AD	L-CA1-body	10.39	<0.001	0.002	0.206
AD	R-GC-ML-DG-head	10.31	<0.001	0.002	0.205
RD	L-Molecular_layer_HP-body	9.97	<0.001	0.003	0.199
MD	L-Molecular_layer_HP-body	9.61	<0.001	0.003	0.194
RD	L-Subiculum-head	9.19	<0.001	0.004	0.187
RD	R-Subiculum-head	9.09	<0.001	0.004	0.185
MD	L-Subiculum-head	8.89	<0.001	0.004	0.182
AD	L-Subiculum-head	8.89	<0.001	0.004	0.182
AD	L-Molecular_layer_HP-body	8.82	<0.001	0.004	0.181
MD	L-CA3-body	8.75	<0.001	0.004	0.179
MD	R-Subiculum-head	8.37	<0.001	0.005	0.173
AD	R-Subiculum-head	8.22	<0.001	0.006	0.171
MD	L-CA1-body	7.67	<0.001	0.008	0.161
RD	L-HATA	7.62	<0.001	0.008	0.160
RD	L-CA3-body	7.29	0.001	0.011	0.154
AD	L-Subiculum-body	7.00	0.002	0.013	0.149
RD	L-Fimbria	6.75	0.002	0.015	0.144
AD	L-Fimbria	6.59	0.002	0.016	0.141
MD	L-HATA	6.59	0.002	0.016	0.141
RD	R-Fimbria	6.34	0.003	0.019	0.137
AD	L-HATA	6.16	0.003	0.021	0.133
MD	L-Subiculum-body	6.13	0.003	0.021	0.133
AD	R-CA1-body	6.12	0.003	0.021	0.133
MD	L-Fimbria	5.99	0.004	0.022	0.130
RD	L-CA1-body	5.99	0.004	0.022	0.130
MD	R-Fimbria	5.64	0.005	0.029	0.124
FA	L-GC-ML-DG-head	5.55	0.006	0.031	0.122
RD	L-Subiculum-body	5.44	0.006	0.033	0.120
MD	R-CA1-body	5.27	0.007	0.037	0.116
AD	R-Fimbria	5.17	0.008	0.039	0.114
AD	R-Subiculum-body	5.04	0.009	0.043	0.112
AD	L-CA4-head	4.97	0.009	0.044	0.111
RD	L-CA4-head	4.91	0.010	0.045	0.109

Type	Outcome	F	P	Global FDR q	Partial η²
RD	R-CA1-body	4.89	0.010	0.045	0.109
FA	R-Molecular_layer_HP-body	4.84	0.010	0.046	0.108
RD	R-GC-ML-DG-body	4.72	0.012	0.050	0.106
MD	L-CA4-head	4.53	0.014	0.058	0.102
RD	R-CA4-head	4.45	0.015	0.061	0.100
FA	L-Subiculum-body	4.01	0.022	0.089	0.091
MD	R-GC-ML-DG-body	3.99	0.022	0.089	0.091
AD	R-CA3-body	3.92	0.024	0.092	0.089
RD	R-CA3-body	3.85	0.025	0.094	0.088
AD	R-GC-ML-DG-body	3.85	0.025	0.094	0.088
RD	R-HATA	3.83	0.026	0.094	0.087
MD	R-CA4-head	3.81	0.026	0.094	0.087
MD	R-CA3-body	3.77	0.027	0.094	0.086
RD	R-Molecular_layer_HP-body	3.76	0.027	0.094	0.086
AD	L-CA4-body	3.71	0.029	0.097	0.085
AD	R-Hippocampal_tail	3.61	0.032	0.105	0.083
FA	R-Subiculum-body	3.49	0.035	0.113	0.080
MD	R-Hippocampal_tail	3.47	0.036	0.113	0.080
RD	R-Hippocampal_tail	3.47	0.036	0.113	0.080
AD	R-CA4-head	3.42	0.037	0.116	0.079
MD	L-Hippocampal_tail	3.34	0.040	0.122	0.077
FA	L-Molecular_layer_HP-head	3.29	0.042	0.127	0.076
RD	L-CA3-head	3.27	0.043	0.127	0.076
AD	L-Hippocampal_tail	3.21	0.046	0.132	0.074
MD	L-CA4-body	3.19	0.046	0.132	0.074
MD	R-Subiculum-body	3.16	0.048	0.134	0.073
FA	L-Presubiculum-head	3.09	0.051	0.138	0.072
MD	R-HATA	3.09	0.051	0.138	0.072
MD	R-Molecular_layer_HP-body	3.07	0.052	0.139	0.071
RD	L-CA4-body	3.02	0.054	0.144	0.070
RD	L-Hippocampal_tail	2.93	0.059	0.154	0.068
AD	R-HATA	2.69	0.074	0.189	0.063
RD	L-GC-ML-DG-body	2.46	0.092	0.232	0.058
MD	L-GC-ML-DG-body	2.27	0.110	0.274	0.054
AD	L-GC-ML-DG-body	2.25	0.112	0.274	0.053
RD	R-Presubiculum-body	2.19	0.118	0.287	0.052
RD	R-Subiculum-body	2.13	0.126	0.301	0.051
FA	R-CA1-head	2.10	0.129	0.305	0.050
Volume	L-fimbria	2.02	0.139	0.324	0.049
FA	R-Subiculum-head	1.91	0.155	0.350	0.046
Volume	L-CA3-body	1.90	0.156	0.350	0.046

Type	Outcome	F	P	Global FDR q	Partial η²
AD	R-Molecular_layer_HP-body	1.89	0.158	0.350	0.045
FA	L-Presubiculum-body	1.88	0.159	0.350	0.045
Volume	R-fimbria	1.88	0.160	0.350	0.045
AD	L-Presubiculum-head	1.86	0.163	0.352	0.044
MD	L-CA3-head	1.83	0.167	0.358	0.044
RD	L-Parasubiculum	1.78	0.176	0.369	0.043
Volume	R-CA1-head	1.77	0.176	0.369	0.043
FA	L-CA4-head	1.71	0.187	0.386	0.041
Volume	R-CA3-body	1.67	0.196	0.400	0.040
FA	L-CA1-head	1.64	0.200	0.405	0.039
RD	R-CA4-body	1.61	0.206	0.412	0.039
AD	R-CA4-body	1.59	0.211	0.416	0.038
Volume	L-presubiculum-head	1.58	0.212	0.416	0.038
FA	R-CA1-body	1.52	0.225	0.436	0.037
RD	L-Molecular_layer_HP-head	1.42	0.248	0.475	0.034
RD	L-CA1-head	1.40	0.252	0.478	0.034
MD	R-Presubiculum-body	1.36	0.262	0.492	0.033
FA	L-Subiculum-head	1.34	0.267	0.496	0.032
MD	R-CA4-body	1.32	0.273	0.502	0.032
AD	R-Presubiculum-body	1.30	0.279	0.504	0.031
Volume	L-parasubiculum	1.30	0.280	0.504	0.032
FA	L-CA1-body	1.28	0.283	0.504	0.031
Volume	L-subiculum-body	1.24	0.296	0.520	0.030
Volume	R-CA3-head	1.23	0.298	0.520	0.030
FA	L-CA3-head	1.22	0.300	0.520	0.030
AD	L-Parasubiculum	1.18	0.311	0.534	0.029
FA	R-Presubiculum-head	1.14	0.326	0.555	0.028
Volume	R-CA4-head	1.07	0.348	0.586	0.026
FA	R-Presubiculum-body	1.06	0.351	0.586	0.026
AD	R-Presubiculum-head	0.99	0.377	0.615	0.024
Volume	R-parasubiculum	0.98	0.379	0.615	0.024
Volume	R-subiculum-body	0.98	0.379	0.615	0.024
FA	R-HATA	0.97	0.382	0.615	0.024
Volume	L-Hippocampal_tail	0.95	0.391	0.622	0.023
Volume	R-Hippocampal_tail	0.94	0.395	0.622	0.023
MD	L-Molecular_layer_HP-head	0.94	0.396	0.622	0.023
RD	R-Parasubiculum	0.91	0.405	0.630	0.022
RD	R-CA1-head	0.88	0.418	0.645	0.022
AD	L-CA3-head	0.82	0.442	0.674	0.020
RD	L-Presubiculum-body	0.82	0.446	0.674	0.020
Volume	L-CA4-body	0.81	0.449	0.674	0.020

Type	Outcome	F	P	Global FDR q	Partial η²
MD	L-CA1-head	0.80	0.452	0.674	0.020
FA	R-CA3-head	0.79	0.458	0.676	0.019
FA	R-Parasubiculum	0.78	0.461	0.676	0.019
MD	L-Presubiculum-head	0.76	0.471	0.676	0.019
Volume	L-CA1-body	0.76	0.473	0.676	0.019
FA	L-HATA	0.75	0.474	0.676	0.019
FA	R-CA4-body	0.74	0.482	0.676	0.018
AD	L-Presubiculum-body	0.74	0.483	0.676	0.018
FA	L-Molecular_layer_HP-body	0.73	0.483	0.676	0.018
Volume	R-CA4-body	0.73	0.487	0.676	0.018
FA	R-CA3-body	0.69	0.503	0.693	0.017
Volume	R-presubiculum-head	0.68	0.510	0.698	0.017
RD	R-CA3-head	0.64	0.529	0.718	0.016
FA	L-Parasubiculum	0.59	0.557	0.747	0.015
FA	R-Fimbria	0.58	0.560	0.747	0.014
AD	L-Molecular_layer_HP-head	0.57	0.567	0.747	0.014
Volume	R-GC-ML-DG-head	0.57	0.568	0.747	0.014
FA	L-Hippocampal_tail	0.56	0.571	0.747	0.014
Volume	L-GC-ML-DG-body	0.56	0.576	0.748	0.014
FA	R-Hippocampal_tail	0.52	0.598	0.772	0.013
MD	R-Presubiculum-head	0.50	0.611	0.783	0.012
Volume	L-CA1-head	0.48	0.623	0.793	0.012
MD	R-CA1-head	0.45	0.642	0.802	0.011
AD	L-CA1-head	0.44	0.642	0.802	0.011
RD	R-Presubiculum-head	0.44	0.643	0.802	0.011
Volume	R-GC-ML-DG-body	0.42	0.657	0.813	0.011
FA	L-CA3-body	0.38	0.683	0.840	0.009
Volume	L-HATA	0.37	0.690	0.842	0.009
Volume	L-subiculum-head	0.36	0.696	0.844	0.009
RD	L-Presubiculum-head	0.35	0.705	0.850	0.009
AD	R-Parasubiculum	0.34	0.712	0.853	0.008
Volume	L-molecular_layer_HP-body	0.32	0.729	0.865	0.008
FA	R-GC-ML-DG-body	0.31	0.732	0.865	0.008
FA	R-Molecular_layer_HP-head	0.28	0.757	0.889	0.007
Volume	L-molecular_layer_HP-head	0.27	0.767	0.895	0.007
Volume	R-molecular_layer_HP-head	0.25	0.780	0.897	0.006
Volume	R-CA1-body	0.25	0.780	0.897	0.006
Volume	R-HATA	0.24	0.784	0.897	0.006
FA	L-Fimbria	0.21	0.807	0.918	0.005
Volume	L-CA4-head	0.19	0.824	0.931	0.005
Volume	R-hippocampal-fissure	0.17	0.840	0.939	0.004

Type	Outcome	F	P	Global FDR q	Partial η²
Volume	R-subiculum-head	0.17	0.843	0.939	0.004
FA	R-CA4-head	0.17	0.848	0.939	0.004
MD	R-CA3-head	0.16	0.852	0.939	0.004
Volume	L-presubiculum-body	0.15	0.857	0.939	0.004
FA	R-GC-ML-DG-head	0.15	0.864	0.941	0.004
FA	L-CA4-body	0.14	0.872	0.944	0.003
MD	R-Molecular_layer_HP-head	0.13	0.879	0.944	0.003
Volume	L-hippocampal-fissure	0.13	0.881	0.944	0.003
Volume	L-CA3-head	0.12	0.890	0.947	0.003
RD	R-Molecular_layer_HP-head	0.11	0.895	0.947	0.003
AD	R-CA3-head	0.10	0.902	0.949	0.003
AD	R-CA1-head	0.09	0.912	0.954	0.002
MD	L-Parasubiculum	0.08	0.921	0.954	0.002
Volume	R-presubiculum-body	0.08	0.925	0.954	0.002
Volume	R-molecular_layer_HP-body	0.07	0.929	0.954	0.002
FA	L-GC-ML-DG-body	0.07	0.936	0.954	0.002
AD	R-Molecular_layer_HP-head	0.05	0.947	0.954	0.001
MD	R-Parasubiculum	0.05	0.948	0.954	0.001
MD	L-Presubiculum-body	0.05	0.949	0.954	0.001
Volume	L-GC-ML-DG-head	0.01	0.988	0.988	0.000

Note. DTI models adjusted for age, sex, and education; volumetric models additionally adjusted for eTIV. Global FDR q values were calculated across all 182 omnibus tests. Values are reported as provided in the verified analysis report.

Table 2. Tukey-adjusted significant pairwise contrasts among outcomes surviving the global FDR screen.

Type	Outcome	Global q	Contrast	Difference	95% CI	Tukey P
AD	L-CA1-body	0.002	HC-PD-MCI	-0.382	-0.630, -0.134	0.001
AD	L-CA1-body	0.002	PD-NC-PD-MCI	-0.350	-0.564, -0.136	<0.001
AD	L-CA3-body	0.002	HC-PD-MCI	-0.386	-0.622, -0.150	<0.001
AD	L-CA3-body	0.002	PD-NC-PD-MCI	-0.323	-0.527, -0.119	<0.001
AD	L-CA4-head	0.044	HC-PD-MCI	-0.135	-0.246, -0.024	0.013
AD	L-Fimbria	0.016	HC-PD-MCI	-0.183	-0.310, -0.055	0.003
AD	L-Fimbria	0.016	PD-NC-PD-MCI	-0.114	-0.225, -0.004	0.041
AD	L-GC-ML-DG-head	0.002	HC-PD-MCI	-0.398	-0.643, -0.153	<0.001
AD	L-GC-ML-DG-head	0.002	PD-NC-PD-MCI	-0.374	-0.586, -0.162	<0.001
AD	L-HATA	0.021	HC-PD-MCI	-0.215	-0.386, -0.045	0.010
AD	L-HATA	0.021	PD-NC-PD-MCI	-0.174	-0.322, -0.027	0.016
AD	L-Molecular_layer_HP-body	0.004	HC-PD-MCI	-0.350	-0.593, -0.106	0.003
AD	L-Molecular_layer_HP-body	0.004	PD-NC-PD-MCI	-0.313	-0.524, -0.103	0.002
AD	L-Subiculum-body	0.013	PD-NC-PD-MCI	-0.330	-0.549, -0.111	0.002
AD	L-Subiculum-head	0.004	HC-PD-MCI	-0.291	-0.495, -0.087	0.003
AD	L-Subiculum-head	0.004	PD-NC-PD-MCI	-0.267	-0.443, -0.090	0.002
AD	R-CA1-body	0.021	HC-PD-MCI	-0.361	-0.665, -0.057	0.016

Type	Outcome	Global q	Contrast	Difference	95% CI	Tukey P
AD	R-CA1-body	0.021	PD-NC-PD-MCI	-0.329	-0.592, -0.066	0.010
AD	R-Fimbria	0.039	PD-NC-PD-MCI	-0.170	-0.308, -0.032	0.012
AD	R-GC-ML-DG-head	0.002	HC-PD-MCI	-0.303	-0.536, -0.069	0.007
AD	R-GC-ML-DG-head	0.002	PD-NC-PD-MCI	-0.359	-0.561, -0.158	<0.001
AD	R-Subiculum-body	0.043	PD-NC-PD-MCI	-0.323	-0.583, -0.063	0.011
AD	R-Subiculum-head	0.006	HC-PD-MCI	-0.386	-0.642, -0.130	0.002
AD	R-Subiculum-head	0.006	PD-NC-PD-MCI	-0.289	-0.510, -0.068	0.007
FA	L-GC-ML-DG-head	0.031	HC-PD-MCI	0.059	0.006, 0.112	0.024
FA	L-GC-ML-DG-head	0.031	HC-PD-NC	0.074	0.019, 0.129	0.005
FA	R-Molecular_layer_HP-body	0.046	HC-PD-MCI	0.044	0.002, 0.086	0.037
FA	R-Molecular_layer_HP-body	0.046	PD-NC-PD-MCI	0.041	0.004, 0.077	0.025
MD	L-CA1-body	0.008	HC-PD-MCI	-0.253	-0.460, -0.045	0.013
MD	L-CA1-body	0.008	PD-NC-PD-MCI	-0.266	-0.445, -0.087	0.002
MD	L-CA3-body	0.004	HC-PD-MCI	-0.274	-0.468, -0.081	0.003
MD	L-CA3-body	0.004	PD-NC-PD-MCI	-0.250	-0.417, -0.083	0.002
MD	L-Fimbria	0.022	HC-PD-MCI	-0.153	-0.267, -0.039	0.006
MD	L-Fimbria	0.022	PD-NC-PD-MCI	-0.101	-0.200, -0.003	0.043
MD	L-GC-ML-DG-head	0.002	HC-PD-MCI	-0.366	-0.589, -0.143	<0.001
MD	L-GC-ML-DG-head	0.002	PD-NC-PD-MCI	-0.338	-0.531, -0.146	<0.001
MD	L-HATA	0.016	HC-PD-MCI	-0.202	-0.358, -0.045	0.008
MD	L-HATA	0.016	PD-NC-PD-MCI	-0.168	-0.304, -0.033	0.011
MD	L-Molecular_layer_HP-body	0.003	HC-PD-MCI	-0.318	-0.538, -0.098	0.003
MD	L-Molecular_layer_HP-body	0.003	PD-NC-PD-MCI	-0.305	-0.495, -0.115	<0.001
MD	L-Subiculum-body	0.021	PD-NC-PD-MCI	-0.262	-0.449, -0.076	0.003
MD	L-Subiculum-head	0.004	HC-PD-MCI	-0.273	-0.469, -0.078	0.004
MD	L-Subiculum-head	0.004	PD-NC-PD-MCI	-0.259	-0.428, -0.090	0.001
MD	R-CA1-body	0.037	PD-NC-PD-MCI	-0.279	-0.503, -0.055	0.011
MD	R-Fimbria	0.029	PD-NC-PD-MCI	-0.165	-0.291, -0.039	0.007
MD	R-GC-ML-DG-head	0.002	HC-PD-MCI	-0.276	-0.487, -0.065	0.007
MD	R-GC-ML-DG-head	0.002	PD-NC-PD-MCI	-0.332	-0.514, -0.149	<0.001
MD	R-Subiculum-head	0.005	HC-PD-MCI	-0.375	-0.625, -0.125	0.002
MD	R-Subiculum-head	0.005	PD-NC-PD-MCI	-0.290	-0.506, -0.074	0.005
RD	L-CA1-body	0.022	HC-PD-MCI	-0.207	-0.406, -0.008	0.040
RD	L-CA1-body	0.022	PD-NC-PD-MCI	-0.229	-0.401, -0.057	0.006
RD	L-CA3-body	0.011	HC-PD-MCI	-0.236	-0.419, -0.053	0.008
RD	L-CA3-body	0.011	PD-NC-PD-MCI	-0.216	-0.374, -0.058	0.004
RD	L-CA4-head	0.045	HC-PD-MCI	-0.110	-0.196, -0.023	0.009
RD	L-Fimbria	0.015	HC-PD-MCI	-0.155	-0.262, -0.048	0.003
RD	L-Fimbria	0.015	PD-NC-PD-MCI	-0.097	-0.190, -0.004	0.038
RD	L-GC-ML-DG-head	0.002	HC-PD-MCI	-0.365	-0.579, -0.152	<0.001
RD	L-GC-ML-DG-head	0.002	PD-NC-PD-MCI	-0.323	-0.508, -0.139	<0.001
RD	L-HATA	0.008	HC-PD-MCI	-0.210	-0.357, -0.062	0.003
RD	L-HATA	0.008	PD-NC-PD-MCI	-0.166	-0.293, -0.038	0.007

Type	Outcome	Global q	Contrast	Difference	95% CI	Tukey P
RD	L-Molecular_layer_HP-body	0.003	HC-PD-MCI	-0.319	-0.535, -0.103	0.002
RD	L-Molecular_layer_HP-body	0.003	PD-NC-PD-MCI	-0.303	-0.490, -0.117	<0.001
RD	L-Subiculum-body	0.033	PD-NC-PD-MCI	-0.230	-0.406, -0.053	0.007
RD	L-Subiculum-head	0.004	HC-PD-MCI	-0.281	-0.473, -0.088	0.002
RD	L-Subiculum-head	0.004	PD-NC-PD-MCI	-0.254	-0.421, -0.088	0.001
RD	R-CA1-body	0.045	PD-NC-PD-MCI	-0.258	-0.469, -0.047	0.013
RD	R-Fimbria	0.019	HC-PD-MCI	-0.149	-0.288, -0.011	0.031
RD	R-Fimbria	0.019	PD-NC-PD-MCI	-0.164	-0.283, -0.044	0.005
RD	R-GC-ML-DG-body	0.050	HC-PD-MCI	-0.141	-0.257, -0.025	0.013
RD	R-GC-ML-DG-head	0.002	HC-PD-MCI	-0.282	-0.483, -0.080	0.004
RD	R-GC-ML-DG-head	0.002	PD-NC-PD-MCI	-0.320	-0.494, -0.146	<0.001
RD	R-Subiculum-head	0.004	HC-PD-MCI	-0.386	-0.630, -0.142	<0.001
RD	R-Subiculum-head	0.004	PD-NC-PD-MCI	-0.290	-0.501, -0.079	0.004

Note. Only pairwise contrasts with Tukey-adjusted P < 0.05 are listed. Difference is defined as the first group minus the second group. The pairwise family comprised the three group contrasts within each globally FDR-significant outcome.

Table 3. Primary partial correlations between MoCA and the 42 FDR-screened DTI outcomes in the pooled PD cohort.

Type	Outcome	Partial r	df	P	95% CI	BH-FDR q	FDR significant
FA	L-GC-ML-DG-head	-0.000	63	0.999	-0.244, 0.244	0.999	No
FA	R-Molecular_layer_HP-body	0.311	63	0.012	0.073, 0.516	0.013	Yes
AD	L-GC-ML-DG-head	-0.401	63	<0.001	-0.588, -0.175	0.002	Yes
AD	L-CA3-body	-0.470	63	<0.001	-0.641, -0.256	<0.001	Yes
AD	L-CA1-body	-0.496	63	<0.001	-0.660, -0.287	<0.001	Yes
AD	L-Subiculum-head	-0.410	63	<0.001	-0.594, -0.184	0.002	Yes
AD	L-Molecular_layer_HP-body	-0.370	63	0.002	-0.563, -0.139	0.004	Yes
AD	L-Subiculum-body	-0.371	63	0.002	-0.564, -0.140	0.004	Yes
AD	L-Fimbria	-0.317	63	0.010	-0.521, -0.079	0.012	Yes
AD	L-HATA	-0.306	63	0.013	-0.512, -0.067	0.014	Yes
AD	L-CA4-head	-0.249	63	0.046	-0.464, -0.005	0.049	Yes
AD	R-GC-ML-DG-head	-0.475	63	<0.001	-0.644, -0.261	<0.001	Yes
AD	R-CA1-body	-0.451	63	<0.001	-0.626, -0.233	<0.001	Yes
AD	R-Subiculum-head	-0.326	63	0.008	-0.528, -0.089	0.010	Yes
AD	R-Fimbria	-0.378	63	0.002	-0.569, -0.148	0.003	Yes
AD	R-Subiculum-body	-0.330	63	0.007	-0.531, -0.094	0.009	Yes
MD	L-GC-ML-DG-head	-0.397	63	0.001	-0.585, -0.170	0.002	Yes
MD	L-Molecular_layer_HP-body	-0.424	63	<0.001	-0.605, -0.201	0.001	Yes
MD	L-Subiculum-head	-0.427	63	<0.001	-0.608, -0.205	0.001	Yes
MD	L-CA3-body	-0.449	63	<0.001	-0.624, -0.230	<0.001	Yes
MD	L-CA1-body	-0.437	63	<0.001	-0.615, -0.216	0.001	Yes
MD	L-HATA	-0.334	63	0.007	-0.534, -0.098	0.009	Yes
MD	L-Subiculum-body	-0.339	63	0.006	-0.539, -0.104	0.008	Yes
MD	L-Fimbria	-0.318	63	0.010	-0.522, -0.080	0.012	Yes
MD	R-GC-ML-DG-head	-0.478	63	<0.001	-0.646, -0.265	<0.001	Yes

Type	Outcome	Partial r	df	P	95% CI	BH-FDR q	FDR significant
MD	R-Subiculum-head	-0.340	63	0.006	-0.539, -0.104	0.008	Yes
MD	R-Fimbria	-0.384	63	0.002	-0.574, -0.154	0.003	Yes
MD	R-CA1-body	-0.414	63	<0.001	-0.598, -0.190	0.002	Yes
RD	L-GC-ML-DG-head	-0.404	63	<0.001	-0.590, -0.177	0.002	Yes
RD	L-Molecular_layer_HP-body	-0.459	63	<0.001	-0.632, -0.242	<0.001	Yes
RD	L-Subiculum-head	-0.445	63	<0.001	-0.622, -0.226	<0.001	Yes
RD	L-HATA	-0.369	63	0.003	-0.562, -0.137	0.004	Yes
RD	L-CA3-body	-0.428	63	<0.001	-0.608, -0.206	0.001	Yes
RD	L-Fimbria	-0.340	63	0.006	-0.539, -0.105	0.008	Yes
RD	L-CA1-body	-0.394	63	0.001	-0.582, -0.166	0.002	Yes
RD	L-Subiculum-body	-0.320	63	0.009	-0.523, -0.083	0.011	Yes
RD	L-CA4-head	-0.223	63	0.074	-0.443, 0.022	0.076	No
RD	R-GC-ML-DG-head	-0.485	63	<0.001	-0.652, -0.273	<0.001	Yes
RD	R-Subiculum-head	-0.358	63	0.003	-0.554, -0.125	0.005	Yes
RD	R-Fimbria	-0.400	63	<0.001	-0.587, -0.173	0.002	Yes
RD	R-CA1-body	-0.394	63	0.001	-0.582, -0.166	0.002	Yes
RD	R-GC-ML-DG-body	-0.242	63	0.052	-0.459, 0.002	0.055	No

Note. n = 68. Pearson partial correlations were adjusted for age, sex, and years of education. eTIV was not included. BH-FDR correction was applied jointly across the 42 P values. The exact verified result was 39/42 FDR-significant associations.

Table 4. Group-adjusted sensitivity partial correlations between MoCA and the 42 DTI outcomes.

Type	Outcome	Partial r	df	P	95% CI	BH-FDR q	FDR significant
FA	L-GC-ML-DG-head	0.260	62	0.038	0.016, 0.476	0.264	No
FA	R-Molecular_layer_HP-body	0.079	62	0.533	-0.170, 0.319	0.678	No
AD	L-GC-ML-DG-head	-0.027	62	0.832	-0.271, 0.220	0.918	No
AD	L-CA3-body	-0.284	62	0.023	-0.495, -0.041	0.264	No
AD	L-CA1-body	-0.273	62	0.029	-0.486, -0.029	0.264	No
AD	L-Subiculum-head	-0.160	62	0.206	-0.391, 0.089	0.457	No
AD	L-Molecular_layer_HP-body	-0.080	62	0.527	-0.320, 0.169	0.678	No
AD	L-Subiculum-body	-0.002	62	0.984	-0.248, 0.243	0.984	No
AD	L-Fimbria	-0.169	62	0.182	-0.398, 0.080	0.457	No
AD	L-HATA	-0.092	62	0.471	-0.330, 0.158	0.678	No
AD	L-CA4-head	-0.083	62	0.513	-0.322, 0.166	0.678	No
AD	R-GC-ML-DG-head	-0.094	62	0.461	-0.332, 0.156	0.678	No
AD	R-CA1-body	-0.299	62	0.017	-0.507, -0.057	0.264	No
AD	R-Subiculum-head	-0.039	62	0.757	-0.283, 0.208	0.868	No
AD	R-Fimbria	-0.155	62	0.220	-0.386, 0.094	0.463	No
AD	R-Subiculum-body	-0.055	62	0.664	-0.297, 0.193	0.796	No
MD	L-GC-ML-DG-head	-0.020	62	0.874	-0.265, 0.227	0.918	No
MD	L-Molecular_layer_HP-body	-0.149	62	0.239	-0.381, 0.100	0.477	No
MD	L-Subiculum-head	-0.192	62	0.128	-0.418, 0.056	0.414	No
MD	L-CA3-body	-0.265	62	0.034	-0.480, -0.021	0.264	No
MD	L-CA1-body	-0.193	62	0.127	-0.419, 0.056	0.414	No

Type	Outcome	Partial r	df	P	95% CI	BH-FDR q	FDR significant
MD	L-HATA	-0.124	62	0.328	-0.359, 0.125	0.598	No
MD	L-Subiculum-body	0.024	62	0.854	-0.224, 0.268	0.918	No
MD	L-Fimbria	-0.168	62	0.185	-0.397, 0.081	0.457	No
MD	R-GC-ML-DG-head	-0.086	62	0.501	-0.325, 0.164	0.678	No
MD	R-Subiculum-head	-0.061	62	0.634	-0.302, 0.188	0.783	No
MD	R-Fimbria	-0.143	62	0.260	-0.376, 0.107	0.496	No
MD	R-CA1-body	-0.234	62	0.062	-0.454, 0.012	0.327	No
RD	L-GC-ML-DG-head	-0.038	62	0.765	-0.281, 0.210	0.868	No
RD	L-Molecular_layer_HP-body	-0.217	62	0.085	-0.439, 0.030	0.372	No
RD	L-Subiculum-head	-0.238	62	0.058	-0.457, 0.008	0.327	No
RD	L-HATA	-0.178	62	0.159	-0.406, 0.071	0.457	No
RD	L-CA3-body	-0.267	62	0.033	-0.481, -0.022	0.264	No
RD	L-Fimbria	-0.209	62	0.097	-0.433, 0.039	0.372	No
RD	L-CA1-body	-0.160	62	0.207	-0.390, 0.089	0.457	No
RD	L-Subiculum-body	0.014	62	0.913	-0.233, 0.259	0.935	No
RD	L-CA4-head	-0.080	62	0.531	-0.319, 0.169	0.678	No
RD	R-GC-ML-DG-head	-0.104	62	0.412	-0.341, 0.145	0.678	No
RD	R-Subiculum-head	-0.094	62	0.459	-0.332, 0.155	0.678	No
RD	R-Fimbria	-0.166	62	0.189	-0.396, 0.083	0.457	No
RD	R-CA1-body	-0.211	62	0.095	-0.434, 0.037	0.372	No
RD	R-GC-ML-DG-body	-0.092	62	0.469	-0.330, 0.157	0.678	No

Note. n = 68. The sensitivity model adjusted for age, sex, years of education, and PD-MCI/PD-NC group membership. BH-FDR correction was applied separately across these 42 sensitivity tests. No association survived FDR correction; the minimum q value was 0.264.
